# Redistribution of Meiotic Crossovers Along Wheat Chromosomes by Virus-Induced Gene Silencing

**DOI:** 10.3389/fpls.2020.635139

**Published:** 2021-02-04

**Authors:** Amir Raz, Tal Dahan-Meir, Cathy Melamed-Bessudo, Dena Leshkowitz, Avraham A. Levy

**Affiliations:** ^1^Department of Plant and Environmental Sciences, Weizmann Institute of Science, Rehovot, Israel; ^2^Department of Plant Science, MIGAL Galilee Research Institute, Kiryat Shmona, Israel; ^3^Bioinformatics Unit, Life Sciences Core Facilities, Weizmann Institute of Science, Rehovot, Israel

**Keywords:** VIGS, meiotic crossover, Met1, DDM1, XRCC2, FANCM, wheat

## Abstract

Meiotic recombination is the main driver of genetic diversity in wheat breeding. The rate and location of crossover (CO) events are regulated by genetic and epigenetic factors. In wheat, most COs occur in subtelomeric regions but are rare in centromeric and pericentric areas. The aim of this work was to increase COs in both “hot” and “cold” chromosomal locations. We used Virus-Induced gene Silencing (VIGS) to downregulate the expression of recombination-suppressing genes *XRCC2* and *FANCM* and of epigenetic maintenance genes *MET1* and *DDM1* during meiosis. VIGS suppresses genes in a dominant, transient and non-transgenic manner, which is convenient in wheat, a hard-to-transform polyploid. F1 hybrids of a cross between two tetraploid lines whose genome was fully sequenced (wild emmer and durum wheat), were infected with a VIGS vector ∼ 2 weeks before meiosis. Recombination was measured in F2 seedlings derived from F1-infected plants and non-infected controls. We found significant up and down-regulation of CO rates along subtelomeric regions as a result of silencing either *MET1*, *DDM1* or *XRCC2* during meiosis. In addition, we found up to 93% increase in COs in XRCC2-VIGS treatment in the pericentric regions of some chromosomes. Silencing *FANCM* showed no effect on CO. Overall, we show that CO distribution was affected by VIGS treatments rather than the total number of COs which did not change. We conclude that transient silencing of specific genes during meiosis can be used as a simple, fast and non-transgenic strategy to improve breeding abilities in specific chromosomal regions.

## Introduction

During meiosis, homologous chromosomes pair and exchange DNA segments. This process, known as homologous recombination (HR), coupled with chromosome pairing, ensures proper segregation, and generates the genetic diversity among gametes. This is the main engine for crop improvement in sexually reproducing crops, hence, high recombination rates would improve breeding capabilities. However, in nature, recombination frequencies are restricted to a narrow range of one to three recombination events per chromosome in each gamete [see (Mercier et al., 2015) for review].

The homologous recombination process starts with the formation of DNA double strand breaks (DSBs) by the SPO11 protein during Leptotene (Keeney et al., 1997). However, only a small portion of the breaks are resolved into crossovers (COs) events. For example in Maize about 20 COs events are resolved from around 500 DSBs in each meiocyte (He et al., 2017). Similarly, in tetraploid wheat about 2.3% of the DSBs resolved as CO events (Desjardins et al., 2020a). Hence, the way DSBs are being repaired is largely responsible for the frequency of COs events. CO formation involves creation and resolution of double Holliday junctions (Whitby, 2005; Gaskell et al., 2007). There are two distinct types of COs, type I and type II, which are outcomes of parallel pathways involving different complexes of proteins (Higgins et al., 2004; Chen et al., 2005; Mercier et al., 2005). Type I COs are subject to CO interference, a process that regulates the distribution of COs along the chromosome, preventing the formation of multiple CO in close proximity (Copenhaver, 2005; Mercier et al., 2005). This is the most prominent CO pathway in plants (Higgins et al., 2004; Hodzic et al., 2004; Wan et al., 2004; Guillon et al., 2005; Mercier et al., 2005; Lhuissier et al., 2007; Falque et al., 2009). Class II pathways which are Mus81-dependent are not subject to CO interference, they represent ∼10% of all CO events in plants and as with class I, class II pathways can also give rise to non-CO events through the resolution of Holliday-like junctions (Mercier et al., 2015). A recent study in tetraploid wheat reports on a ratio of 85% class I versus 15% class II events (Desjardins et al., 2020a). Another HR pathway that gives rise only to non-CO events is the synthesis-dependent strand annealing (SDSA) mechanism (Rubin and Levy, 1997; Allers and Lichten, 2001; Hunter and Kleckner, 2001; Börner et al., 2004). Research in *Arabidopsis* mutants led to the identification of three different pathways controlling recombination using either: *FANCM* (Crismani et al., 2012; Girard et al., 2014), *RECQ4A* and *RECQ4B* together with *TOP3*α and *RMI* (Séguéla-Arnaud et al., 2015, 2017), or *FIGL1* (Girard et al., 2015). An increase in CO rate by a factor of up to 3.6 was reported in the *fancm* mutant (Crismani et al., 2012) and a 1.5 and 6.2 fold increase in the *top3*α and *recq4a-recq4b* mutants, respectively (Hartung et al., 2007; Séguéla-Arnaud et al., 2015). In these experiments most of the additional COs were of the type II CO pathway. Furthermore, the *figl1 recq4* and *fancm recq4* double mutants showed about 10 fold increase in recombination rate reaching an unprecedented amount of 12 COs per *Arabidopsis* chromosome (Fernandes et al., 2018). Increase in COs events in *recq4* and *fancm* mutants was also found in different crops such as rice, tomato, pea, and turnip mustard (Blary et al., 2018; Mieulet et al., 2018; Fayos et al., 2019) suggesting that these genes serve as universal meiotic anti-CO genes which suppress mainly type II COs. Another anti-CO gene is the RAD51 paralog XRCC2. Serra et al. (2013) found a 50% increase in recombination rate in the *xrcc2 Arabidopsis* mutant compared to wild type.

Double strand breaks and crossovers are not uniformly distributed along the chromosome, instead, they tend to concentrate in hotspots (Mézard, 2006; Mézard et al., 2007; Pan et al., 2011; Smagulova et al., 2011). In wheat, most of the CO hotspots are found in the sub-telomeric regions while the centromeric and peri-centromeric regions which occupy large portions of the chromosome, show very low recombination rate (Avni et al., 2014; Choulet et al., 2014). What turns certain chromosomal regions as hotspots is not fully understood, however, mounting evidence suggest the involvement of epigenetic markers. For example H3 histone lysine 4 trimethylation (H3K4me3) and chromatin accessibility were found to correlates with DSB hotspots in yeast and mouse (Berchowitz et al., 2009; Borde and de Massy, 2013). In human and mouse, the key determinant for recombination hotspot – the PRDM9 protein – is a histone methyltransferase which target 13 bp long CCN repeat motif (Baudat et al., 2010; Myers et al., 2010). Although in plants a paralog for the *PRDM9* gene is still to be found, three short motives were found to be enriched in *Arabidopsis* and maize CO hotspots – CCN-repeat, CTT-repeat and A-rich motif (Shilo et al., 2015; He et al., 2017). Analysis of the epigenetic landscape around these motives in *Arabidopsis* and maize revealed a peak of H3K4me3, H3K27me3, and H2A.Z histone modification, as well as negative peak of nucleosome occupancy and CG methylation (Choi et al., 2013; Shilo et al., 2015; He et al., 2017). Since epigenetic markers may influence the occurrence of a CO, manipulating genes related to these markers may change the distribution or the rate of recombination events along the chromosome. In plants, maintenance of DNA methylation depends on the context where CG is methylated by *DNA METHYLTRANSFERASE 1 (MET1*) (Kankel et al., 2003), while CHG and CHH are methylated by *CHROMOMETHYLASE*s (*CMT2* and *CMT3*) (Lindroth, 2001). In addition, experiments in *Arabidopsis thaliana* showed that *DECREASE IN DNA METHYLATION 1* (*DDM1*) protein is involved in methylation maintenance of all cytosine contexts by releasing the wrapped DNA from the nucleosome (Lyons and Zilberman, 2017). Experiments in *Arabidopsis* showed that down regulating cytosine methylation through mutations in *DDM1* or *MET1*, correlates with an increase in the rate of CO in euchromatin but not in pericentromeric heterochromatin regions (Melamed-Bessudo and Levy, 2012; Mirouze et al., 2012; Choi et al., 2013). Underwood et al. (2018) showed that mutating the CHG DNA methyltransferase gene *CMT3* in *Arabidopsis*, led to increase in meiotic recombination rate even at the peri-centromeric regions.

Considering the above experiments, it seems possible to achieve recombination increments in wheat and maybe to affect CO localization, by mutating anti-CO and DNA methylation genes. Transformation and genome editing in wheat, as well as selection of homozygous and multiple mutants by TILLING is difficult and time-consuming with very low efficiency due to both its polyploid nature and the technically challenging transformation protocols. The most commonly used methods for cereal transformation is either *Agrobacterium*-infection or particle bombardment. Both methods rely on tissue culture procedures where the treated tissue (usually embryos) generates calli cells that can be regenerated into a transgenic plant. This procedure can take up to several month and transformation rates are low. Moreover, the end product is a Genetically Modified Organism (GMO) which is not accepted by regulators in many countries. Recently, the lab of Caixia Gao greatly improved wheat transformation procedures, and managed to perform a knockout mutation in wheat by delivering components of the CRISPR-Cas9 system transiently using either Ribonucleotides-Proteins (RNPs) or mRNA (Zhang et al., 2016; Liang et al., 2017; Sánchez-León et al., 2018) which resulted in non-GMO mutants. However, the efficiency of this procedure is lower and the chances to mutate all alleles is even lower, thus it is labor intensive and not shortening the timescale. Simultaneous knock-out of all alleles of a specific gene in the same plant is possible. However, as the number of alleles increase in polyploid plants (as many as six alleles in bread wheat) the chance to obtain all the mutations in the same plant decreases, forcing at least one round of hybridization. Furthermore, knocking-out a gene is in many cases too drastic and leads to sterility, especially when targeting a housekeeping gene, as was shown in a *ddm1* knockout of tomato and maize plants (Corem et al., 2018; Fu et al., 2018). In cases like this, silencing approach such as microRNA or siRNA can be used. However, this still requires tissue culture transformation and results in GMO plants. Using a Virus Induced Gene Silencing (VIGS) system as a gene silencing method is an alternative to the traditional iRNA/siRNA cassettes. This system offers the advantages of fast and simple cloning stage followed by an easy and highly efficient infection. Another important feature of this method is a transient effect, which lasts 2 to 4 weeks, enabling the plant to grow normally at the end of the treatment. This was successfully used in wheat for both basic and applicative researches (Bennypaul et al., 2012; Lee et al., 2015). Moreover, VIGS treatment were successfully applied to manipulate meiotic-specific processes in wheat and *Arabidopsis* (Bhullar et al., 2014; Calvo-Baltanás et al., 2020; Desjardins et al., 2020b).

In this work, we used VIGS to silence meiotic anti-CO genes as well as DNA methylation genes during meiosis to study the effect of specific genes on meiotic recombination and to increase the rate of CO events in various regions of wheat chromosomes. The ability to manipulate COs is important for plant breeders, in particular in crosses with exotic germplasm, in which the CO rate is low, or when trying to break linkage between genes or bring new allelic variation to genes that are located in pericentromeric regions. VIGS offers the possibility to alter recombination rates without any genetic modification such as mutagenesis or transgenesis. We have tested the effect of *MET1, DDM1, XRCC2, RecQ4, and FANCM* genes on HR rates in tetraploid wheat in progeny of a fertile hybrid between wild emmer wheat, the direct progenitor of domesticated tetraploid wheat (WEW, var. Zavitan) and durum wheat (var. Svevo) where both parents have a well-characterized genome (Avni et al., 2017; Maccaferri et al., 2019; Zhu et al., 2019). We show that silencing of *MET1* and *DDM1* during meiosis led to redistribution of HR events in euchromatic regions while silencing of *XRCC2* resulted in redistribution of HR in both euchromatic and heterochromatic regions. Other genes tested had no effect on meiotic recombination.

## Materials and Methods

### Plant Material

Seeds were germinated in a growth chamber for 4–5 weeks on a long day set up of 16 h of light and a temperature of 18°C at night and 20°C during the day. Plants were then moved to a greenhouse for the rest of the experiment and were grown under the same temperature regime. For hybrid formation, “Svevo” flowers were emasculated at heading stage and bagged for 4–5 days, followed by pollination with “Zavitan” fresh pollen. Spikes were kept bagged until seeds were fully developed.

### VIGS Cloning and Propagation

All VIGS procedures were adapted from Lee et al. (2015) with minor changes. In short, a 250–400 bp segment was designed for each gene using the si-Fi (siRNA Finder)^1^ software, based on the “Zavitan” WEW transcriptome. Anti-sense sequences were amplified from Zavitan genome using specific primers (Supplementary Table 1) and cloned into the BSMV RNAγ vector pCa-γbLIC (Yuan et al., 2011) via ligation-independent cloning (LIC) and transformed into *Agrobacterium tumefaciens* strain GV3103 as described (Lee et al., 2015). Four weeks old *Nicotiana benthamiana* leaves were co-infiltrated with a mix of *Agrobacterium tumefaciens* strains carrying BSMV RNAα, RNAβ, and RNAγ together in 1:1:1 ratio. Infected leaves were collected 5 days post infection and either stored at −80°C for later use or were used immediately for wheat infection. Non-infiltrated leaves were collected 8 days post infection to verify systemic infection ability of the virus. To that end, total RNA was purified using Nucleospin RNA Plant kit (MACHEREY-NAGEL) followed by cDNA synthesis with Verso cDNA synthesis kit (Thermo Fisher Scientific). Viral presence was verified using primers from the virus genome and the specific insert (Supplementary Table 1).

### VIGS Infection

*Nicotiana benthamiana* infiltrated leaves were grounded under liquid nitrogen in 10 mM potassium phosphate pH 7.0 containing 2% w/v Celite© 545 AW (Sigma–Aldrich) in a ratio of 1.5 ml per 1 g of leaf tissue. Crude extracts were used to infect wheat leaves of 15 different spikes using two methods simultaneously: rubbing the leaf with two fingers and injecting the leaf with needle less syringe in two locations along the leaf. Time of infection was 2–3 weeks before meiosis, typically on the third or fourth leaf. Infected plants were sprayed with a mist of water and covered with plastic bags for the night. Plants were allowed to grow until spikes were dry and seeds were collected separately from each infected tiller.

### qPCR

**FIGURE 1:**
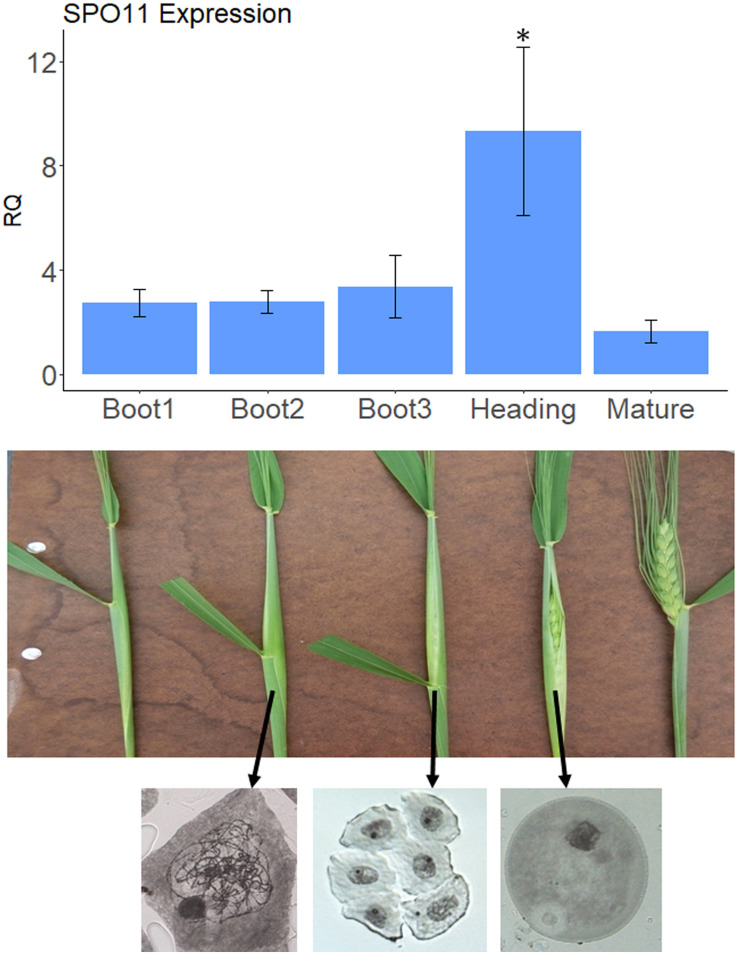
*SPO11* expression at different developmental stages. The upper panel shows relative expression levels of *SPO11* as determined by qPCR, during five developmental stages of the spike, shown in the bottom panel for cv. Svevo. The reference gene was *Actin*. Bars represent SE, the number of replica, *N* = 3, Asterisk designate significant differences from the Mature stage (*p* < 0.05). The bottom panel shows meiotic stage analysis by Acetocarmine staining of male meiocytes taken from the middle spikeletes at different physiological stages. Arrows show origin of stained meiocyes. Left, zygotene; Middle, tetrad; Right, young pollen.

For analysis of *SPO11* expression, anthers from three different spikes were gently collected from 3 to 4 spikelets at the middle of the spike for each booting or maturation stage (Figure 1). For analysis of the VIGS effect, anthers from each of the 15 infected spikes were gently collected from 3 to 4 spikelets at the middle of the spike between boot2 and boot3 stages (Figure 1). Total RNA was purified using Nucleospin RNA Plant kit (MACHEREY-NAGEL) followed by cDNA synthesis with Verso© cDNA synthesis kit (Thermo Scientific). qPCR analysis was done in a StepOnePlus© real time system (Applied Biosystems). Each reaction contained 5 μl FAST Sybr (Applied Biosystems), 1 μl mixed primers (Supplementary Table 2) at 2 μM, and 2 μl of sample containing 40–50 ng cDNA. Relative expression was calculated using *Actin* as internal normalization gene (Bhullar et al., 2014). Note that alternative normalization genes for wheat meiosis (not used here) were recently reported and should be used in future works (Garrido et al., 2020).

### Markers Design

In order to design simple PCR markers we aligned the sequence of chromosome 1A of “Svevo” and “Zavitan” and we screened for InDels (20–200 bp) which are easy to distinguish on a simple agarose electrophoresis gel. The InDels were detected by an in house developed pipeline that utilized public tools. Specially, initially alignment was done between Zavitan chromosome and the Svevo genome (160802_Svevo_v2_pseudomolecules.1.fasta) using the program NUCmer from MUMmer (version 3.23; parameters : -maxmatch -l 100 -c 500) (Kurtz et al., 2004). The output out.delta was analyzed with the program Assemblytics (parameter: 200)^2^. The bed output variants_between_alignments was filtered (using awk) to contain InDels that are between 20 and 200 bases long that align to chromosome 1A of Svevo. We found more than 2000 such InDels. Annotation of the InDel region was added using Homer script annotatePeaks.pl^3^. The 150 base sequence surrounding the InDel was extracted using bedtools getfasta^4^. In addition, to ensure that the certain sequence of Svevo does not have an homologous region in Svevo or and additional homologous region in Zavitan genome, blastn was run (version 2.5.0, parameters: -outfmt 7 -max_target_seqs 1) against the relevant genomes in which the InDel regions were masked by running bedtools program maskfasta.

We choose 12 deletions (in “Svevo” compared to “Zavitan”) spreading all along the chromosome. Primers were carefully designed for chromosome-specific amplification, namely sequences of both “Zavitan” and “Svevo” chromosome 1A, but not of the homoeologous chromosome 1B nor from paralogous loci (Supplementary Table 3). DNA was purified from the first or second leaves of seedlings using Nucleospin DNA Plant© kit (MACHEREY-NAGEL). PCR reactions were done in 96 plates in total volume of 15 μl using Hy-Taq ready mix© (Hy-Labs, Israel) and products were analyzed by gel electrophoresis.

### GBS Libraries Preparation and Analysis

Genotyping-By-Sequencing libraries were prepared following the protocol by Poland et al. (2012). Libraries were sequenced by Illumina NextSeq 550 mid-output using 150 base-pairs single-end kits. Reads were mapped to a “combined” genome containing the Zavitan WEW_v2.0 genome (Zhu et al., 2019) and the Svevo.v1 genome (Maccaferri et al., 2019) using *bwa-mem* (Li, 2013). Mapped reads were converted to binary alignment map (BAM) format and filtered for high quality (>30), uniquely mapped and perfect matched using SAMtools package (Li et al., 2009). Zavitan and Svevo-specific reads served to build each “combined” genome. We found an average of 32,000 to 64,000 markers per chromosome, namely parent-specific reads. Each pair of chromosomes was divided into identical number of ∼1 Mb bins and the number of filtered reads was calculated for each bin using BEDtools (Quinlan and Hall, 2010). For each pair of matching bins (from Zavitan and/or Svevo) the number of mapped reads was summed together. For each bin, the ratio between Zavitan reads and Svevo reads was calculated. Each bin was then re-calculated as the mean ratio of the surrounding 15 bins. A bin was genotyped as homozygous if the calculated ratio was higher than 0.9 (Zavitan) or lower than 0.1 (Svevo), otherwise it was considered as heterozygous. Bins with less than 10 reads were ignored. COs were assigned to regions where bins changed from one genotype to another. Double COs were ignored if the distance between them was less than 8 Mb for subtelomeric regions or less than 70 Mb for pericentric regions. We applied this analysis on libraries of Zavitan and Svevo as well. Between 2 and 3% of the Bins were not consistent with parental genotypes and were removed from the progeny analysis.

### Statistics

Data analysis and statistics were done in the R environment. In most cases, Wilcoxon test was used as significance test, except for recombination rate where the Chi square test was used.

### Cytological Analysis

Staging of meiosis was done using contrast-phase microscopy: spikes were fixed in Carnoy’s solution (3 ethanol: 2 chloroform: 1 glacial acetic acid) and anthers were squashed with Acetocarmine (Feldman, 1966).

## Results

Our goal was to silence genes that are putative suppressors of recombination during meiosis, when recombination between homologs occurs. Meiosis in wheat occurs during early booting stage. In order to determine the optimal stage to check for the silencing effect we sampled anthers from three different spikes at each booting stages as well as heading and mature spikes and checked the expression levels of *SPO11* as a meiotic marker. As shown in Figure 1, the level of expression of *SPO11* in anthers starts to increase already at Boot1 stage (in comparison with non-meiotic mature anthers) reaching the highest levels at heading, and going down after emergence of the spike (Mature stage). To be on the safe side we decided to sample anthers between Boot2 and Boot3 in order to test silencing of our target genes, considering also the fact that zygotene occurs during Boot2 stage as seen by chromosome staining (Figure 1).

### Virus-Induced Gene Silencing of Recombination Suppressors

**FIGURE 2:**
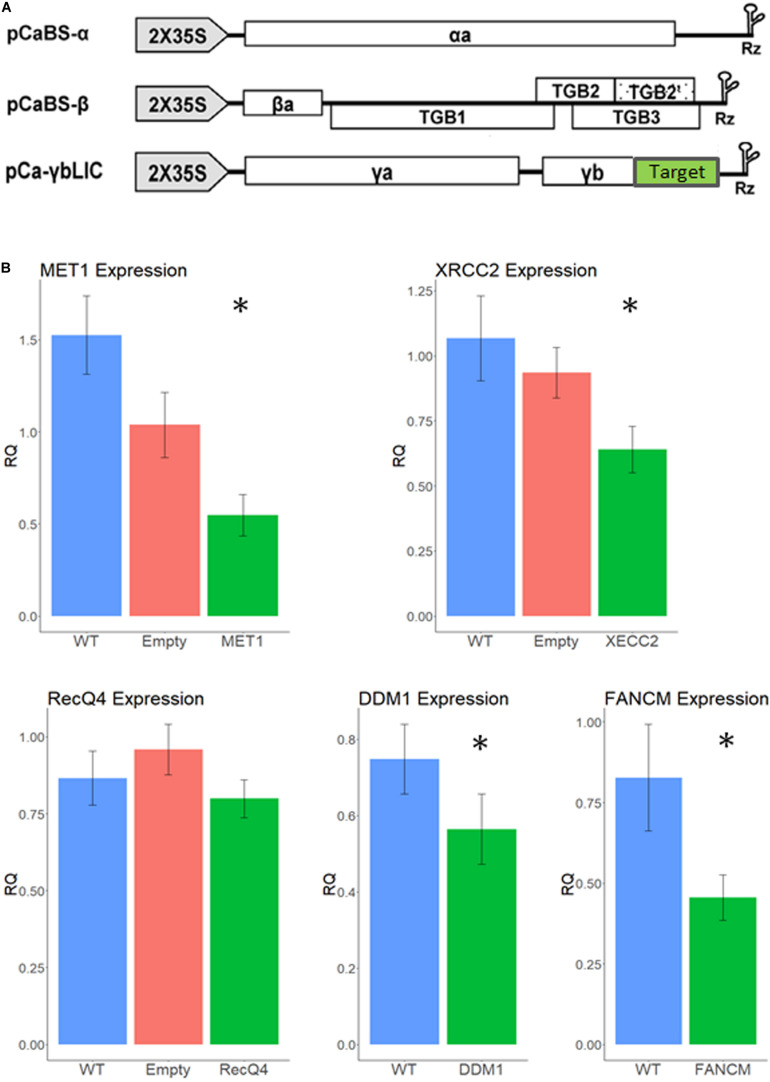
Relative expression levels of recombination suppressor genes after infection with different VIGS treatments. **(A)** VIGS constructs. Each of the three BSMV sub-genomes was cloned into the pCass4-Rz binary vector under the 35S promoter. Target (green box) correspond to the gene of interest sequence (Adapted from Yuan et al., 2011). Vectors were introduced into *N. benthamiana* leaves for viral propagation and extracts from these leaves were used for wheat infection. **(B)** Silencing effect by VIGS treatments. Normalized relative expression is shown on the *Y* axis for each gene studied; WT, un-infected plants. Empty – infection with empty virus. Asterisk designate significant difference from WT (*p* < 0.05, *N* = 15). Error bars represent SE.

We have infected 15 tillers of F1 cv. Svevo x cv. Zavitan hybrid plants, with the recombinant BSMV (Figure 2A) 2 to 3 weeks before anthesis, usually on the third or fourth leaf, using both needle-less infiltration and the rubbing method (Lee et al., 2015). While designing the VIGS constructs, we carefully choose sequences that show high similarity between the two homoeologous allele as well as between the two parents of the hybrid. Accordingly, qPCR primers were designed from highly conserved sequences in the mRNA to match all four possible alleles. Thus, a lower expression level in the qPCR test reflects the total silencing effect of all four alleles of each gene. Anthers from three different spikelets, each from a different tiller, were sampled at Boot2 stage to measure expression levels of each gene by qPCR and assess the silencing effect. As shown in Figure 2A VIGS worked well on *MET1, DDM1, FANCM*, and *XRCC2* genes, reducing their expression level between 65 and 24% (*p* < 0.05) compared to WT plants (Figure 2B). The empty vector treatment showed some non-significant reduction in gene expression, possibly due to the stress effect of the virus infection. There was no significant reduction in expression of *RecQ4*, therefore we did not pursue further analyses with this gene which, originally, was a lead candidate (Mieulet et al., 2018).

### Fertility in VIGS-Treated Plants

In order to check whether the silencing treatment of F1 cv. Svevo x cv. Zavitan hybrid plants had a deleterious effect on the gametes or the developing seeds, we counted the number of F2 seeds in the treated F1 spikes (Supplementary Figure 1). Silencing of *FANCM* or *DDM1* showed significant reduction in seeds number, reaching 5–7 seeds per spike compared to 19 seeds in the WT. The other treatments showed only mild, but non-significant reduction.

### Crossover Rate in VIGS-Derived Seeds

**FIGURE 3:**
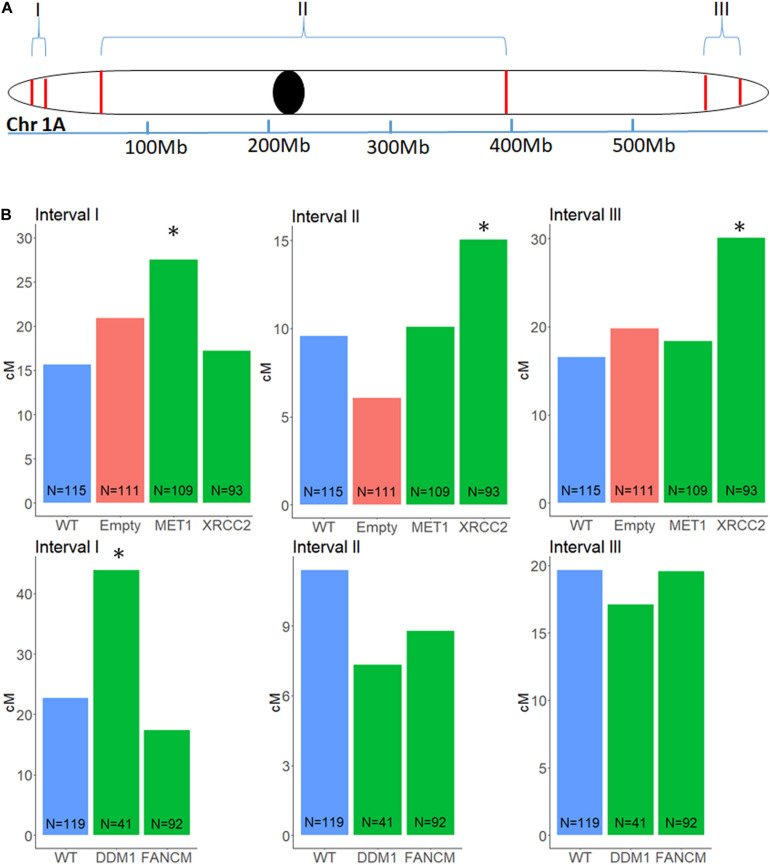
Genetic distance in a F2 (Zavitan x Svevo) population, between three pairs of markers on chromosome 1A, after VIGS treatment of F1 plants. Markers were selected from a list of InDels between Zavitan and Svevo chromosomes. **(A)** Schematic map of the three intervals measured for changes in genetic distance. I, left arm sub-telomeric region; II, peri-centric region; III, right arm sub-telomeric region. **(B)** Effect of *VIGS* for *MET1*, *XRCC2*, *DDM1, FANCM* and their untreated WT F2 plants and empty vector control. locations of transition between genotypes correspond to a CO event. The number of plants in each population N, is marked at the base of the column. Asterisk designate significant difference from WT (*p* < 0.05).

To analyze recombination rate in the F2 progenies of F1 cv. Svevo x cv. Zavitan hybrid plants that underwent VIGS and of negative controls that were treated with an empty vector, we developed a series of InDel markers, that are easy to screen for, through a whole genome comparison of the “Zavitan” and “Svevo” genomes. We focused on chromosome 1A, where we choose 12 InDels markers along the chromosome. All markers have a 100–200 bp larger “Zavitan” product, so that a simple gel-electrophoresis was sufficient for genotyping. We selected three pairs of markers with genetic distance of 9 to 22 cM: one for each sub-telomeric region and another one spanning the pericentric region (Figure 3A). We used these three intervals to measure recombination rates. Progenies of F1 plants treated with MET1-VIGS as well as DDM1-VIGS showed increment in recombination of 76 and 94%, respectively, at the left arm in sub-telomeric region but not in the other intervals. XRCC2-VIGS progenies showed an increment of 82% in the right arm sub-telomeric region and, interestingly, a 57% increase in the pericentric region (Figure 3B). The treatment with FANCM-VIGS showed no significant changes in recombination. In order to check the total number of recombination events in chromosome 1A, we used 12 InDel markers along the chromosome to identify all events in each progeny. We found no overall increase of recombination events in any of the treatments (Supplementary Figure 2) but rather redistribution of crossover sites.

### Genotyping by Sequencing (GBS)

**FIGURE 4:**
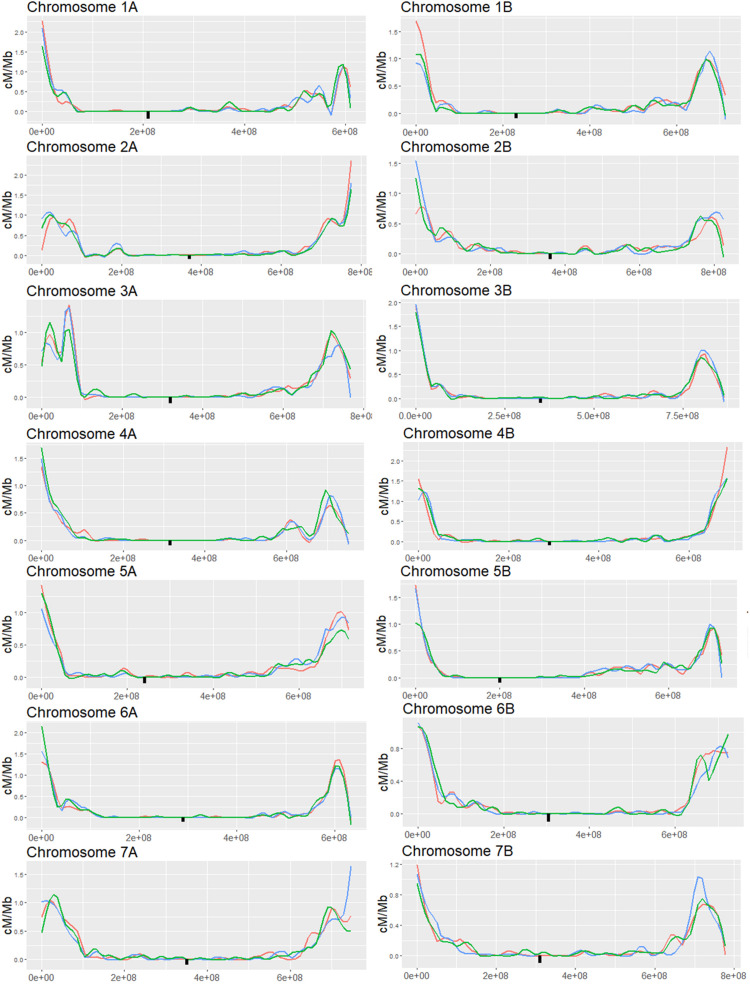
Genome wide analysis of F2 (Zavitan x Svevo) populations derived from F1 plants which were treated by either MET1-VIGS or XRCC2-VIGS during meiosis or untreated (WT). COs were analyzed in F2 progenies by GBS method followed by NGS Illumina sequencing. Chromosomes were divided into 1 Mb bins which were genotyped according to the ratio of mapped reads. Genetic distance in centi-Morgans per mega base pairs (*Y* axis) is shown along each chromosome (*X*-axis represent bp in Zavitan chromosomes). Blue, WT control; Red, MET1-VIGS treatment; Green, XRCC2-VIGS treatment; Black square, centromere position.

To follow-up on the results of the markers analysis, we expanded the analysis to the whole genome with higher resolution (32,000 to 64,000 markers per chromosome) to better characterize the silencing effect on CO distribution. We choose to focus on the MET1-VIGS and XRCC2-VIGS treatment since these treatments showed significant changes in CO events and minor loss in seeds number. We used GBS-NGS approach (Poland et al., 2012) to genotype the same progenies populations used for the above low-resolution markers analysis. Reads were mapped to a combined “Zavitan”-“Svevo” genome and collapsed into ∼ 1 Mb bins (598 to 851 bins per chromosome). On average, we found 62.9 reads per bin while in the pericentric region we found 38.8 reads per bin and in the left and right subtelomeric regions the average reads count was 102.7 and 73.9, respectively. Bins were genotyped as homozygous when more than 90% of its mapped reads belonged to one of the parents. COs were assigned to the junctions between adjacent bins differing in their genotype. To validate the consistency between the GBS analysis and the markers analysis, we computed the genetic distance of the three intervals in chromosome 1A and found high correlation between the GBS analysis and the markers results (Supplementary Figure 3). As in previous studies on Zavitan-Svevo hybrids (Avni et al., 2014), we found that most of the CO events were concentrated in hotspots at the subtelomeric regions while the pericentric regions showed a very low rate of recombination (Figure 4). Changes in CO rates following VIGS treatments, were observed in both the subtelomeric and pericentric regions. However, these changes did not show a consistent pattern of either increase or decrease in CO rates but rather a redistribution of the hotspots along the chromosome. Indeed, the total number of COs per chromosome was not affected by the treatment (Supplementary Figure 4B), however, there were several significant local effects in both pericentric and subtelomeric regions where CO rate was either increased or decreased at a specific locus compared to WT plants.

**FIGURE 5:**
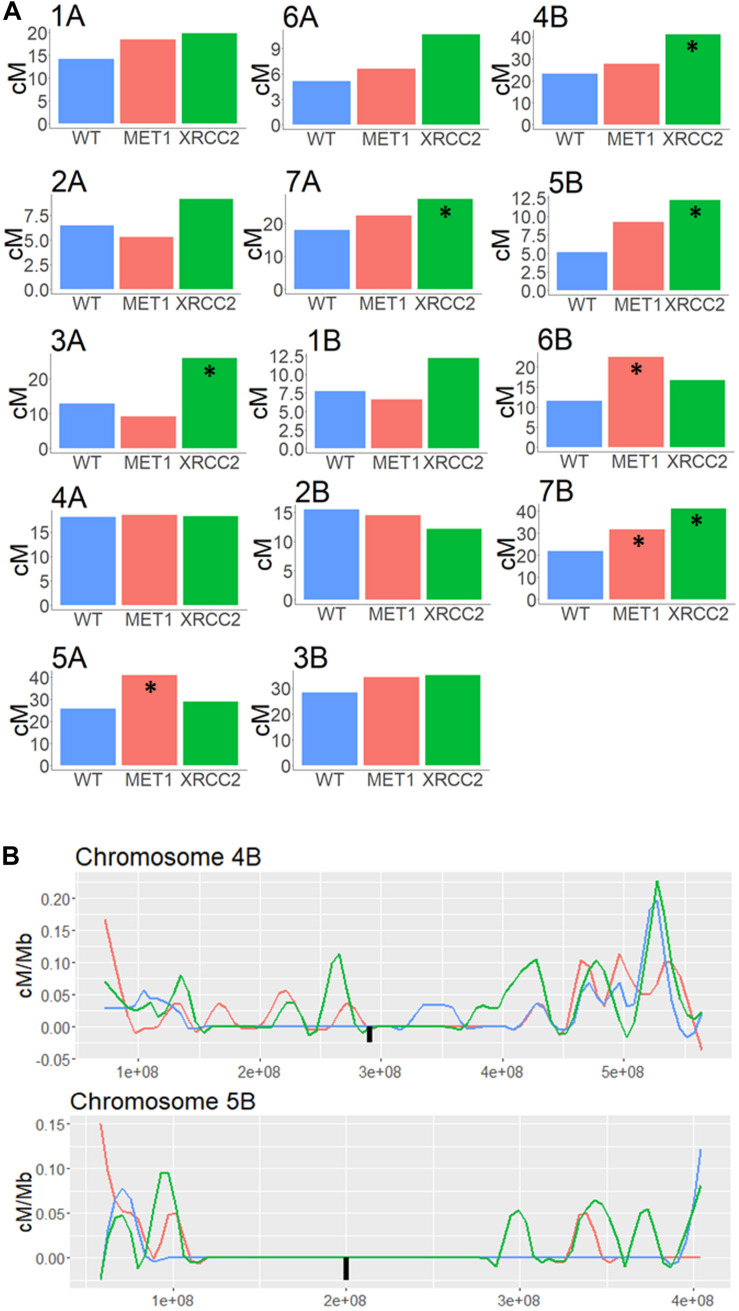
Genetic distance analysis in Pericentric regions of F2 (Zavitan x Svevo) populations derived from F1 plants treated by either MET1-VIGS or XRCC2-VIGS during meiosis compared to untreated F1s (WT). **(A)** Genetic distance (centi-Morgans) of pericentric region in each chromosome. Asterisk marks treatments significantly different from untreated WT (chi-squared test, *p* < 0.05). **(B)** High resolution analysis of genetic distance (centi-Morgans/Mb) in pericentric regions of chromosome 4B and 5B in F2 (Zavitan x Svevo) populations derived from F1 plants treated by MET1-VIGS (Red, *N* = 87), XRCC2-VIGS (Green, *N* = 82) or untreated WT control (Blue, *N* = 89). The *X*-axis represent bp in Zavitan chromosomes. The Black square represents the centromere position.

Regions surrounding the centromere, which showed less than 0.1 cM/Mb in the untreated WT population were considered as pericentric. We summed all the CO events in each chromosome and checked whether either of the VIGS treatment led to a significant increased recombination rate in this area. Interestingly, silencing *XRCC2* led to a significant increase of between 51% and 136% in five of the chromosomes (Figure 5A). In addition, *MET1* silencing led to a significant increase of 44 to 93% in three of the chromosomes. As shown in examples of chromosomes 4B and 5B (Figure 5B), some of these increases are a result of COs which occurred in the close proximity of the centromere, whereas in the WT population we found virtually zero COs in these regions. On average, over all chromosomes, there was a significant enhancing effect on COs of 45 and 25% in pericentric regions when silencing *MET1* or *XRCC2*, respectively (*N* = 14, *p* < 0.05) (Supplementary Figure 4A), however, this was mostly due to effects originating from specific chromosome as shown in Figure 5A.

**FIGURE 6:**
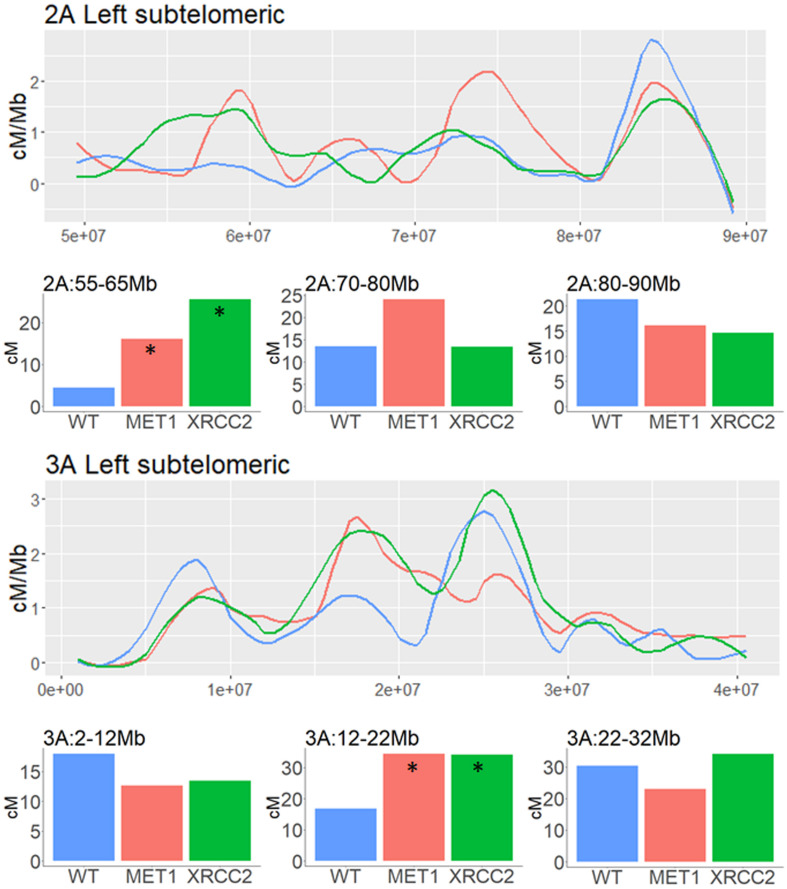
Analysis of genetic distance in subtelomeric regions of three different chromosomes of F2 (Zavitan x Svevo) populations derived from F1 plants which were treated by either MET1-VIGS or XRCC2-VIGS during meiosis or untreated (WT). Lines show the Genetic distance in centi-Morgans per Mb along subtelomeric regions (*X*-axis represent bp in Zavitan chromosomes). Bars show the genetic distances in centi-Morgans in three 10 Mb intervals from each subtelomeric region. Asterisk indicate a significant difference from WT (chi-squared test, *p* < 0.05). Blue, WT control (*N* = 89); Red, MET1-VIGS treatment (*N* = 87); Green, XRCC2-VIGS treatment (*N* = 82).

In subtelomeric areas, the VIGS effects were very variable and context-dependent. As shown in the examples of subtelomeric regions of chromosomes, 2A and 3A, local increase and decrease in CO events can be found in close proximity when comparing both VIGS treatment to the WT control in these regions (Figure 6).

## Discussion

In this work we have used the VIGS method developed by Lee et al. (2015) to silence various meiotic anti-CO and DNA methylation genes. By careful timing of the infection, we were able to reduce the transcripts levels of most of these genes in a transient manner at the stage spanning meiosis. A weakness of the VIGS method applied to meiosis is that it is not possible to accurately control and measure the degree of silencing in the meiotic cells. Likewise, it is not clear why a target like *RecQ4* was not silenced by VIGS in this experiment. Nevertheless, the benefits, as described below, compensate for this weakness. The transient nature of this method is advantageous over a stable gene silencing or knock out mutation for several reasons: it is non-transgenic and can be applied easily to any hybrid; it is transient so that if deleterious, the gene silencing effect is constrained in time; it is dominant and enables stacking of genes compared to the lengthy process of recessive mutations and double mutants production (especially important in polyploid hybrids); when affecting meiotic recombination its effect is transmitted to the next generation. For example, *MET1* and *DDM1* participate in the maintenance of DNA and chromatin methylation state and play a key role in maintenance of genome stability through suppressing of transposable elements (Ito and Kakutani, 2014; Paszkowski, 2015), thus permanent deficiencies in their activity may lead to a mutator effect and eventually to sterility. Moreover, even if not sterile, these mutants reduce plant fitness, and therefore once their effect has been achieved one has to “return” to wildtype to obtain a desired crop. Likewise, a full knockout of *DDM1* or *FANCM* might limit their use in breeding programs as suggested by the reduction in fertility observed by silencing.

### Silencing DNA Methylation Genes

On the basis of studies showing increased recombination in *Arabidopsis* mutants (Melamed-Bessudo and Levy, 2012; Mirouze et al., 2012; Choi et al., 2013), we silenced *MET1* and *DDM1* genes during meiosis. In spite of the mild reduction in *DDM1* expression, we observed a drastic reduction in fertility of 74%, which may be a result of genome instability caused by enhanced activity of transposable elements. These findings are in line with the sterility found in a *ddm1* tomato, maize and rice mutants (Corem et al., 2018; Fu et al., 2018). Silencing the wheat homologs of *DDM1* and *MET1*, led to a mix trend in the subtelomeric regions, where dramatic increase and decrease in COs were found in the same chromosome and in some cases in the same subtelomeric region, implying a change in hotspots strength rather than absolute change in recombination rates (Figure 6). Remarkably, increases in recombination tend to occur in a region that is already a hotspot in WT, suggesting that hot becomes hotter, and next to it, possibly due to genetic interference, a decrease in recombination is seen (Figure 6). Since the rate of COs in the pericentric area is so low, we assessed the genetic distance of the whole areas which span 291–568 Mb around the centromere. We found some strong enhancing effects in three chromosomes by MET-VIGS treatment (Figure 5A) but a milder average effect throughout the genome (Supplementary Figure 4A). Effects were stochastic in the pericentric region with new “lukewarm-spots” being formed but remaining 10–20 fold lower than hotspots in subtelomeric regions. It might be that regions which were completely silenced in WT plants became slightly more accessible when *MET1* was silenced. A report in *Arabidopsis*, by Underwood et al. (2018), showed that mutating the CHG DNA methyltransferase gene *CMT3* in *Arabidopsis*, led to increase in meiotic recombination rate in some peri-centromeric regions. Hence, it might be of interest to use VIGS to silence the wheat *CMT3* during meiosis. These results also highlight the efficacy of the approach in bypassing the expected lethality of these mutants in wheat.

### Silencing Meiotic Anti CO Genes

In this study, we have applied VIGS to different anti-CO genes during meiosis. Unfortunately, the expression of the leading candidate genes, *RecQ4* homeologs, could not be reduced by VIGS. Silencing was achieved for *FANCM* and *XRCC2* but only *XRCC2* had significant effects on CO rates. A reduction in fertility was also found when silencing *FANCM* but no effects were observed on CO rate. Fernandes et al. (2018) reported that a *fancm* mutant has no effect on recombination in Col/Ler hybrid *Arabidopsis*, as opposed to the significant increase in recombination in the col parent reported by Crismani et al. (2012). Since our experiment was done on a hybrid of wild emmer and durum wheat, the lack of effect of FANCM-VIGS on CO might be due to either hybridity or to inter-species differences. The best results in all parameters were obtained when silencing the *XRCC2* gene. In this treatment, no significant reduction in fertility was found while increase in recombination was observed not only in sub-telomeric but also in the peri-centromeric region.

### Genome-Wide

The total number of CO events along the chromosomes using the markers or the GBS analysis showed no differences between WT or VIGS treatments. This implies that the distribution rather than the amount of crossovers was affected as a result of the treatments. Nevertheless, if even a small proportion of the total COs were “moved” toward the pericentric region, or another cold region including genes of interest, VIGS may improve our ability to break linkages between genes or to introduce new allelic variation to pericentric regions.

## Conclusion

In this work we examined a new way to enhance recombination events in progenies of a hybrid tetraploid wheat. We used the VIGS method to silence meiotic anti CO genes and DNA methylation genes during meiosis. We found a redistribution of recombination events in euchromatic and heterochromatic regions when *MET1*, *DDM1*, and *XRCC2* were silenced. Applying this method on more genes (such as *CMT3*) or silencing few genes in parallel as was done in *Arabidopsis* may further enhance meiotic recombination. We showed that this method can be used as a simple fast and non-GMO tool to modify the recombination landscape and enhance variation in certain regions for more efficient plant breeding.

## Data Availability Statement

The data presented in the study are deposit in the Sequence Read Archive (SRA) at https://www.ncbi.nlm.nih.gov/sra/, accession numbers PRJNA691681, PRJNA691573, and PRJNA691706.

## Author Contributions

AL and AR designed the research and wrote the manuscript. AR performed the research. DL did InDels analysis. TD-M helped with GBS calibration and data analysis. TD-M and CM-B helped with library preparation and NGS sequencing. All authors contributed to the article and approved the submitted version.

## Conflict of Interest

The authors declare that the research was conducted in the absence of any commercial or financial relationships that could be construed as a potential conflict of interest.

## Footnotes

1 http://labtools.ipk-gatersleben.de/

2 http://www.ncbi.nlm.nih.gov/pubmed/27318204

3 http://homer.ucsd.edu/homer/ngs/annotation.html

4 https://bedtools.readthedocs.io/en/latest/
