# Supplementary material for: Redistribution of Meiotic Crossovers Along Wheat Chromosomes by Virus-Induced Gene Silencing
Source: Front Plant Sci. 2021 Feb 4;11:635139. doi: 10.3389/fpls.2020.635139 (PMC7890124; doi:10.3389/fpls.2020.635139)
Supplement: Supplementary file 2 [file Data_Sheet_2.docx]

**Supplementary data**

**Table S1**: primers used for BSMV vectors construction of different gene of interest.

| **Primer name** | **Sequence** | **Annealing temperature (˚c)** |
| --- | --- | --- |
| DDM1-3 | AAGGAAGTTTAATGCGGAAGGCAGATCTAAGT | 60 |
| DDM1-4 | AACCACCACCACCGTTGAAATGGAAGGGAAAACAGA |  |
| FANCM-1 | AAGGAAGTTTAATCCCTTTACGCAAAAGGTTC | 68 |
| FANCM-2 | AACCACCACCACCGTTTTCACCGGATGAGGATGAT |  |
| MET1-3 | AAGGAAGTTTAACGTTCAATGCAATAGTGCTACC | 60 |
| MET1-4 | AACCACCACCACCGTTGGGATACCAGGTTCGTTTT |  |
| RecQ4A-1 | AAGGAAGTTTAATCCCAGGGTCCATTCATATC | 60 |
| RecQ4A-2 | AACCACCACCACCGTCAAACTAGCTTTTTGCCCAGA |  |
| XRCC2-1 | AAGGAAGTTTAAAAATTTCGTTCCTGGCTTCA | 68 |
| XRCC2-2 | AACCACCACCACCGTCAAGTCCCAGCTGCTCCTC |  |
| 2235.F * | GATCAACTGCCAATCGTGAGTA | 60 |
| 2615.R* | CCAATTCAGGCATCGTTTTC |  |

*Primers from BSMV genome for constructs verification (Lee et al., 2015).

**Table S2**: Primers used for qRT-PCR analysis on silenced plants.

| **Primer name** | **Sequence** | **Annealing temperature (˚c)** |
| --- | --- | --- |
| ACTIN RTfw* | TGTGCTTGATTCTGGTGATGGTGTG | 60 |
| ACTIN RTbk* | CGATTTCCCGCTCAGCAGTTGT |  |
| DDM RT2fw | ACACCTCGTGCAAACCAACC | 60 |
| DDM RT2bk | TGTTGCAAAACCCGCTTCC |  |
| FANCM RT3fw | GTGAGCAAGATCAAACGAGTATCC | 60 |
| FANCM RT3bk | CCTTCTGTTTCCAACATAATGCTC |  |
| MET1 RT2fw | AGCAAGCTGGTGTGTCATTT | 60 |
| MET1 RT2bk | CCATGATTGCCTTTAAAATCAC |  |
| RecQ4 RTfw3 | GATGGATTGAGAAAGATGTGACC | 60 |
| RecQ4 RTbk3 | ATCATGACGGTTCTTCTTGAC |  |
| SPO11 RTfw | TACCCCTCCATGTTCGTAGA | 60 |
| SPO11 RTbk | ATCACCAAACCTTTTGCCAC |  |
| XRCC RT3fw | ATAGATCGTGCTCTTCAACCTAC | 60 |
| XRCC-RT3bk | CACCAAAATAGGTTGAAGTTGC |  |

* (Bhullar et al., 2014)

**Table S3**: Primers used for markers analysis on chromosome 1A.

| **Primer name** | **Sequence** | **Annealing temperature (˚c)** |
| --- | --- | --- |
| M1-fw | TGTATGAAGAGAATCGGGTTG | 59 |
| M1-bk | CGCGGAGAAAGGAGAAGAA |  |
| M1a-fw | CTCGGCTCTCACACTGCTAA | 63 |
| M1a-bk | GCGGCATCAATGGAGGACG |  |
| M1c-fw | GTGACTTCCCGAGTACAAACC | 60 |
| M1c-bk | CCACACCTATCTTCCCAAAGA |  |
| M2-fw | TCTGGATGCATGGTTATTCCCT | 60 |
| M2-bk | AAAGGTCACAGGCAGTTTCAT |  |
| M4-fw2 | CGGCCACGGAATAACCTGC | 60 |
| M4-bk2 | AGTGAGTCCCAGTCCCAGGTG |  |
| M6-fw | GGAGTTCTGTGTCCTGTCTACTAGG | 65 |
| M6-bk | TTGGCACTGTCATTTGGCTC |  |
| M7-fw | AAGTCACTGTTACAATTTCTGTTCAC | 59 |
| M7-bk | GCAATGTTTGAACTCATGAACAG |  |
| M7b-fw | GGAGGCGCGCGATGGTG | 68 |
| M7b-bk | AGGGCATGCTTGTCCTCATC |  |
| M8-fw | TAGGGAATCTTCAATGGCAACC | 59 |
| M8-bk | ATCTGGGGTGTTACAATGCC |  |
| M8b-fw | CGTAGCCCAACTACGTAAACTG | 60 |
| M8b-bk | CATATTGAGCCCCTTCATGG |  |
| M8c-fw | CATTTTCGTATGGCGAGCTT | 61 |
| M8c-bk | GTTTTTGAGGGGTGTCCAAA |  |
| M10-fw | GCGCGTATAAAGTTAGTTCTTAGAGG | 60 |
| M10-bk | TTAACTCAATTGCGGATGTTG |  |


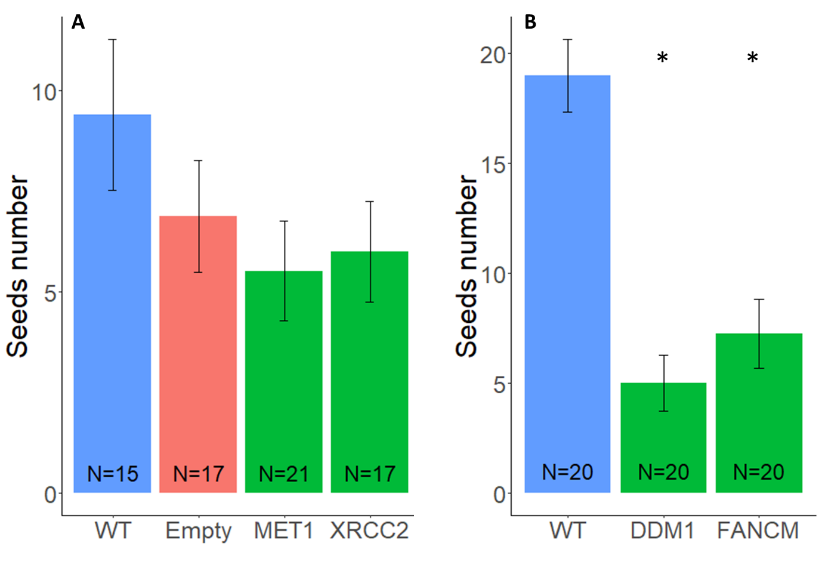


**Figure S1**: Mean seeds number per spike in VIGS treated spikes of F1(Zavitan x Svevo) plants. Seed number is shown as a % of WT. A – First experiment. B – Second experiment. Asterisk designate significant difference from WT (p<0.05). Bars represent SE.


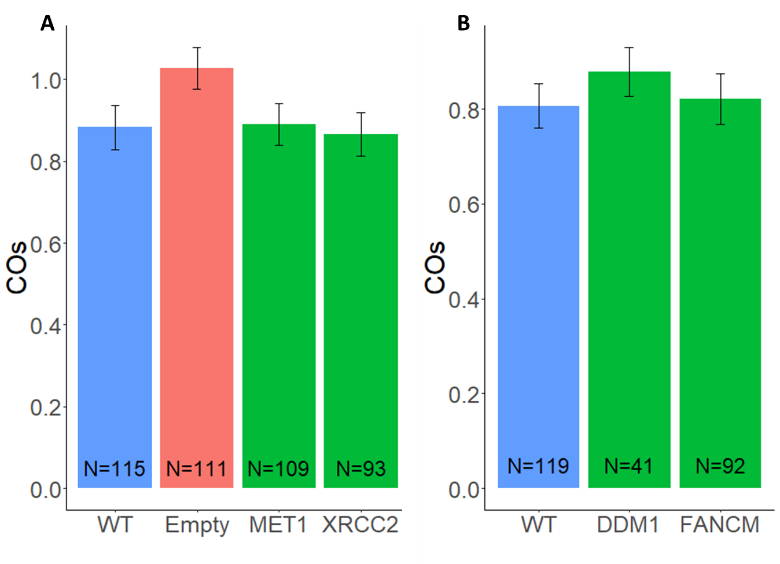


**Figure S2**: Mean number of cross-over events along all intervals of chromosome 1A after VIGS treatment in F2 (Zavitan x Svevo) populations derived from F1 plants treated by either MET1-VIGS, XRCC2-VIGS, DDM1-VIGS or FANCM-VIGS during meiosis compared to untreated F1 plants (WT) or VIGS-treated with an empty vector (Empty). A – First experiment. B – Second experiment. Bars represent SE.


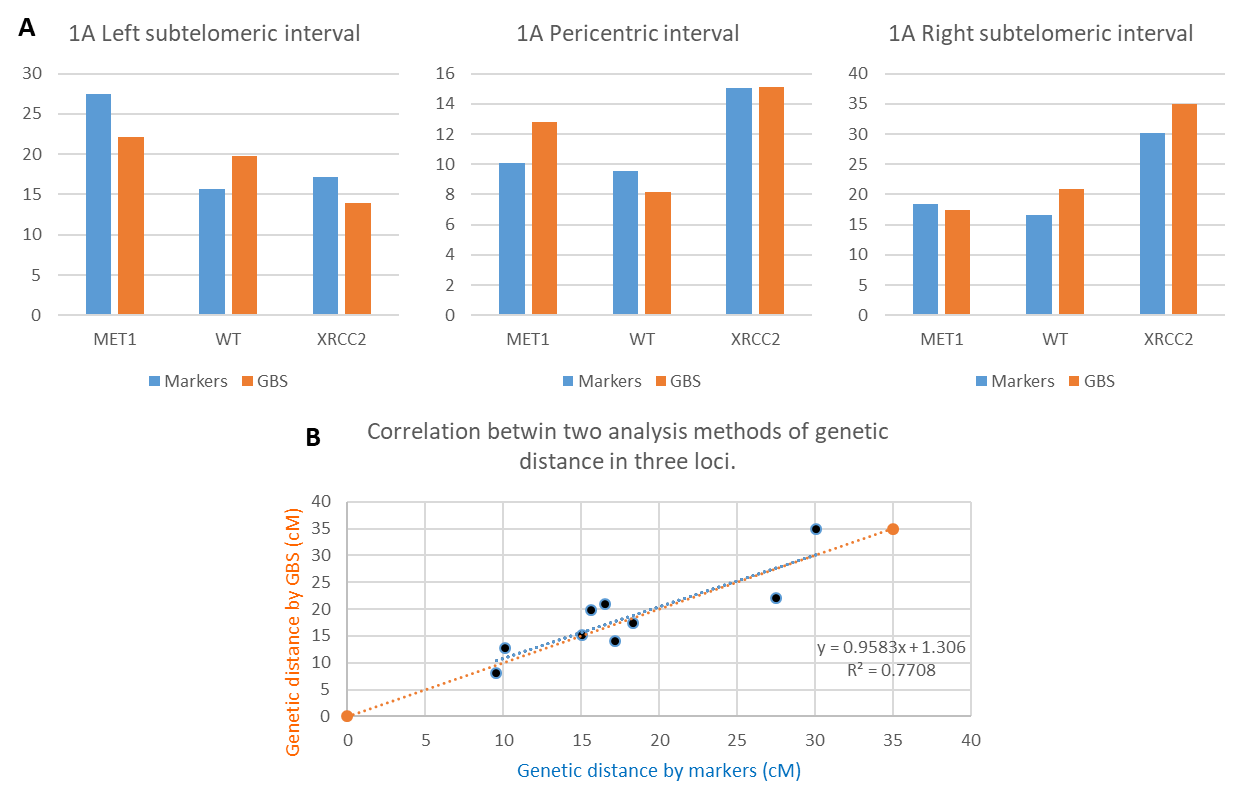


**Figure S3**: Correlation between markers and GBS analysis methods of genetic distances in three intervals of chromosome 1A. A – Genetic distance of three intervals in chromosome 1A, after VIGS treatment was analyzed by PCR markers (orange) and GBS method (blue). B – Correlation between genetic distances analyzsis by either PCR markers or GBS.


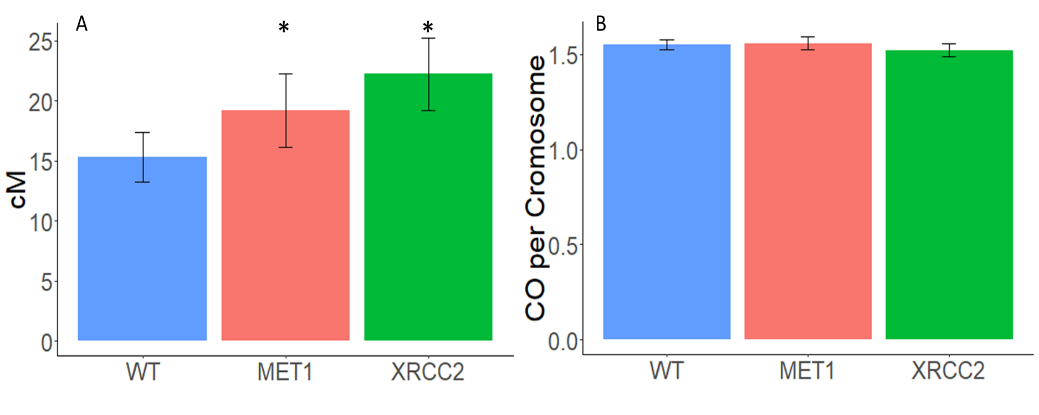


**Figure S4** – Mean genetic distance comparison of 14 chromosomes in WT control and MET1 or XRCC2 VIGS treatments. A – Genetic distance of all pericentric regions. B – Number of CO per chromosome. Asterisk – significantly differ from WT (wilcoxon test, p<0.05).

Bhullar, R., Nagarajan, R., Bennypaul, H., Sidhu, G. K., Sidhu, G., Rustgi, S., et al. (2014). Silencing of a metaphase I-specific gene results in a phenotype similar to that of the Pairing homeologous 1 (Ph1) gene mutations. *Proc. Natl. Acad. Sci. U. S. A.* 111, 14187–14192. doi:10.1073/pnas.1416241111.

Lee, W. S., Rudd, J. J., and Kanyuka, K. (2015). Virus induced gene silencing (VIGS) for functional analysis of wheat genes involved in Zymoseptoria tritici susceptibility and resistance. *Fungal Genet. Biol.* 79, 84–88. doi:10.1016/j.fgb.2015.04.006.
